# Guest editorial note on integrated care pathways: a basic tool for Triple aim improvement of healthcare by collaborative local action

**DOI:** 10.5334/ijic.1123

**Published:** 2012-12-18

**Authors:** Torben Larsen

**Affiliations:** CAST, University of Southern Denmark

The organization of the care process pursuing a Triple aim improvement of healthcare is a common interest of clinicians, healthcare managers and policy-makers in this period of financial shortage especially as part of Obamacare in the US [1]. The Triple aim is: 1) better perception of care by the patients e.g. by better clinical continuity, 2) better professional effectiveness e.g. by better planning and coordination of the whole patient pathway and at the same time 3) reducing or at least controlling the cost of care e.g. readmissions. Care pathways, clinical pathways or integrated care pathways have so far been used as synonymous terms for a new approach to such improved organization of health care. The term originates from industrial processes in the late 1990s in the US such as production re-engineering and lean management as explained in the theoretical paper by Guus Schrijvers in the oncoming Special Issue of *IJIC* on Pathways [2] from which a number of studies deserve a special attention.

Clinical pathways have been defined and discussed by the European Pathway Association (EPA) as a response to the growing complexity of care [3]. According to EPA, a care pathway is “A complex intervention for the mutual decision-making and organization of predictable care for a well-defined group of patients during a well-defined period”. An example of the need of clinical pathways is the following: to decide on a specific drug for a stroke patient is from the point of view of organization fairly simple as it is exclusively rooted in medical science personalized in the higher ranking physician. However, the formation of a stroke unit requires multidisciplinary collaboration, e.g. the involvement of different therapists, other specialists and the patient which changes the organizational focus from a vertical to a horizontal perspective. Defining characteristics of clinical pathways are:

- An explicit statement of the goals and key elements of care based on evidence, best practice and patient expectations
- Facilitation of the communication and coordination of roles by sequencing the activities of the multidisciplinary care team, patients and their relatives
- Documentation, monitoring and evaluation of variances and outcomes
- Identification of relevant resources

Not all care pathways implicate integrated care. We claim that the term integrated care pathway (ICP) should refer especially to pathways integrated across various health care settings, e.g. hospitals and municipal health services. Hence, multidisciplinary collaboration within a single ward is not in itself an ICP. In the oncoming special issue of *IJIC* [2], a set of ICPs—developed as part of the European Seventh Framework project, Homecare 222954—serve to complement the integration across sectors using the home of the patient as an overlapping station for a specialized outreaching hospital team and general community services as represented by integrated homecare (IHC) for patients suffering from stroke, heart failure and COPD, respectively. As illustrated in Figure 1 the discharge pathway for patients suffering from such chronic diseases is traditionally managed by general medical practitioners. The special challenge of IHC is to organize a direct horizontal, inter-organizational collaboration by a patient pathway integrating specialized medical care and general healthcare. In IHC the role of the GP is relatively unaffected.

The first example of IHC is the early home-supported discharge (EHSD) for stroke patients presented by Langhorne et al. [2]. It is remarkable that the outcome of EHSD was only demonstrated when the Cochrane Trialists moved focus from traditional hospital-based functional indices as Barthél Index or Functional Independence Measure (FIM) to broader hard endpoints as ‘Poor Outcome’ (Aggregate of deaths and disabled by 6 months follow-up). This illustrates in a nut-shell the core effect of IHC: it is not so much derived from specific somatic training but more from improved psychological coping. The next example of IHC addresses patients suffering from heart failure (HF) as presented by Jaarsma et al. [2]. Also regarding HF is the core issue better mental coping rather than specific somatic training as seen from the fact that the primary outcome is a reduction in the risk of all-cause-readmission rather than specific readmission for HF. The third and last example of better coping by IHC addresses COPD presented by Alonso et al. [2]. In this case is it nearly self-evident that the anxiety component of exacerbations is a critical cause for readmissions. So, self-management education in the handling of exacerbations in the home-setting in combination with the security provided by a hotline to a well-known nurse explains the reduction in the risk of readmissions by IHC for COPD.

As a forerunner of [2] the referenced papers on IHC an independent review by Tummers et al. of the economic evidence on integrated care for stroke patients was published in the IJIC July–September 2012 issue [4]. Three different models of integrated care for stroke patients were reviewed: 1) Stroke units are more expensive than conventional care, but improve health outcomes; as an alternative 2) Home-based Stroke rehabilitation is unlikely to give cost-savings, but improves health outcomes; 3) Early home-supported Discharge (EHSD) as a kind of integrated middle model is the only model both reducing costs and as demonstrated by Langhorne et al. [2] improves health outcomes, too. So, the health economic dominance of IHC (Win-Win-situation for both patients and society) as concluded by the Homecare-project is directly supported by the independent study of Tummers et al. [4].

Other ICP than IHC may benefit patients, too! A survey among 120 patients on changes in lifestyle habits since attending a new Hepatitis C clinic is reported by Horwitz et al. [2]. Respondents who had attended the clinic for more than 6 months by this new pathway were significantly more likely to commence Hepatitis C treatment over the next 5 years.

It takes new knowledge and skills of staff inexperienced in such collaborative working to understand the factors at stake, e.g. self-management of horizontal coordination of multidisciplinary skills. Development of generic service delivery strategies seems difficult as local implementation projects will be greatly influenced by their local cultural, financial and political contexts. However, EPA has presented a strategy for the proper use of clinical pathways. The idea is that a complete pathway product should have four distinct and interrelated levels, see Figure 2 [3]. The IHC pathways are described in practical, non-normative guides focusing on: 1) What´s the typical pathway phases and activities? 2) What’s the evidence? 3) For which patients and with which outcomes? 4) With which content and dose? 5) What staff competences are required? The practical IHC guides presented above are examples of ‘Model pathways’.

A follow-up to Homecare 222954 focuses the local operation of IHC which differs substantially from the model pathway:

- In high-income countries with tax-financed health care as in Northern and Western Europe the primary local challenge is to establish good collaborative relations between hospitals and the surrounding municipalities
- In Southern Europe lacking municipal welfare-resources, the local challenge seems to be to establish good hospital-based homecare-units to advance from a relative low quality to the level of best international practice in one developmental step.
- In private insurance-based healthcare systems in Central Europe, e.g. Germany and the Netherlands, the local challenge seems to be to establish sufficiently competent providers of integrated care outside the hospital.

Further development and consolidation of ICPs require studies at the patient level. The prospective assignment of pathways may be studied by qualitative interviews to improve patient experiences while retrospective studies of completed pathways may be useful for economic rationalization e.g. giving feedback to the model level on barriers to change. A study on “Informal work and formal plans: articulating the active role of patients in cancer trajectories” by Dalsted et al. [2] exemplifies such qualitative feedback to the formal health care system. Such study is reported to help to integrate care by a more holistic and personalized approach as a complementary service around the formal pathways across settings. The outcome is reported as more active patients.

We hope that the studies presented in the oncoming special issue [2] may help to accelerate the development of ICPs towards a Triple Aim improvement of health care by local projects whatever they may be driven by hospitals, municipalities or private providers.

## Figures and Tables

**Figure 1.**
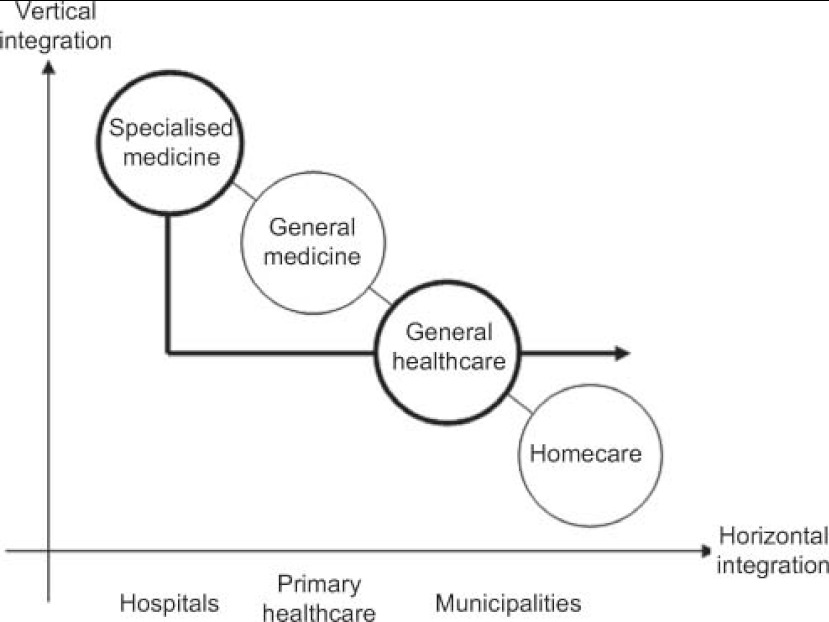
The IHC patient pathway.

**Figure 2.**
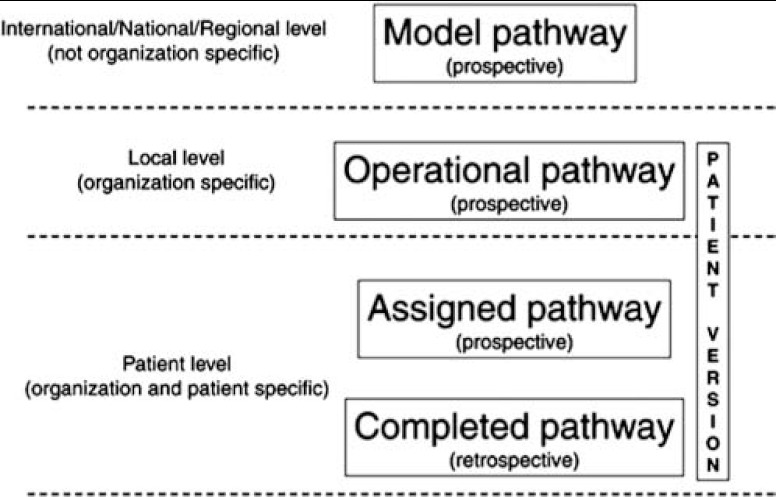
Four distinct interactive levels of pathway development [3].
